# Supplementary material for: Impact of Isoniazid Preventive Therapy on Tuberculosis incidence among people living with HIV: A secondary data analysis using Inverse Probability Weighting of individuals attending HIV care and treatment clinics in Tanzania
Source: PLoS One. 2021 Jul 13;16(7):e0254082. doi: 10.1371/journal.pone.0254082 (PMC8277069; doi:10.1371/journal.pone.0254082)
Supplement: Attachment — Submitted filename: Rebuttal Letter_Response to Reviewers.pdf [file pone.0254082.s006.pdf]

Dr. Werner Maokola

Ministry of Health, Community Development, Gender, Elderly and Children

National AIDS Control Program

Government City-Mtumba

Afya Road

P. O. Box 743

40478 DODOMA

June 15, 2021

Katalin Andrea Wilkinson, PhD

Academic Editor

PLOS ONE

Re: Responses to comments made on research titled: "Impact of Isoniazid Preventive Therapy on Tuberculosis incidence among people living with HIV; a secondary data analysis using Inverse Probability Weighting of individuals attending HIV care and treatment clinics in Tanzania".

Thank you very much for taking time to reviewing my manuscript and providing constructive ideas in order to improve it.

I have gone through the comments raised by the editor and the reviewers; the following are the responses to the comments. The number lines quoted in the rebuttal letter refer to clean version of the revised manuscript:

1. **Tables of results** are now included as part of the main manuscript and individual files have been removed.
2. **Laboratory protocols:** The study involved secondary data analysis from routine HIV program in Tanzania. No laboratory procedures were conducted as such for this research apart from normal routine hospital procedures for Tuberculosis diagnosis.

3. **List of references:** The list of references has been reviewed; incomplete/retracted references have been removed from the list of references. Duplicated references were removed.
4. **Availability of data:** Data used for the study has been made available. It is available in the research repository of London School of Hygiene and Tropical Medicine, United Kingdom. The data can be accessed using the following link: https://www.lshtm.ac.uk/sites/default/files/2017-06/Tanzania%20CTC%20Documentation.pdf

5. **Line 100: "TB infection"** has been replaced by LTBI. Moreover**,** the whole manuscript has been reviewed and changes have been made; TB infection is now referred as Latent TB infection (LTBI) to distinguish it from TB disease (Lines 50 in the clean revised manuscript).
6. **Line 106: C**larification on the meaning of the exposure of interest "IPT" and "non-IPT" has been included (Lines 105-107 in the revised clean manuscript)
7. **Lines 121-123 and Lines 130-132**: Relevant changes have been made (Lines 127-129 in the revised clean manuscript)
8. **Abstract:** A statement rather than a recommendation has been included in the background section of the abstract (Lines 11-13 in the revised clean manuscript). The objective has been made smart (Lines 14-15 in the revised clean manuscript).
9. **Discussion: Lines 239 -244:** Revision has been made (Lines 236-239in the clean manuscript)
10. **Discussion: Explanation on lower TB incidence in 2012 and in private health facilities**: Explanation on lower TB incidence in 2012 and in private health facilities has been included (Lines 273-285 in the revised clean manuscript).
11. **Funding**: The study was funded through SEARCH (Sustainable Evaluation through Analysis of Routinely Collected HIV data) Project under Bill and Melinda Gates Foundation grant number OPP1084472 entitled "Using routinely collected public facility data for program improvement in Tanzania, Malawi and Zambia.". Routine HIV data management is Tanzania is managed collaboratively by the Government of the Republic

of Tanzania and the Government of the United States of America through President's Emergency Plan for AIDS Relief.

Attached in this letter find the responses of the comments from reviewers.

Sincerely,

Dr. Werner Maokola
