# Supplementary material for: Impact of Isoniazid Preventive Therapy on Tuberculosis incidence among people living with HIV: A secondary data analysis using Inverse Probability Weighting of individuals attending HIV care and treatment clinics in Tanzania
Source: PLoS One. 2021 Jul 13;16(7):e0254082. doi: 10.1371/journal.pone.0254082 (PMC8277069; doi:10.1371/journal.pone.0254082)
Supplement: Attachment — Submitted filename: Responses to the editor.docx [file pone.0254082.s002.docx]

Responses to reviewer’s comments:

1. Please remove your figures from within your manuscript file, leaving only the individual TIFF/EPS image files. These will be automatically included in the reviewer’s PDF.

**Thank you very much for the comment. We have removed all the figures from within the main manuscript and sent separately.**

2. Thank you for including your ethics statement: "The study used de-identified routinely collected HIV data. Permission to use data was obtained from National Institute of Medical Research (NIMR) Tanzania by signing data transfer agreement with the Ministry of Health, Community Development, Gender, Elderly and Children (MoHCDGEC).".

**Thank you very much for the comment. The research for the submitted manuscript was approved by Kilimanjaro Christian Medical College (KCMUCo) Institutional Review Board (IRB) which granted the ethical clearance certificate number 2287. Moreover, the parent project known as SEARCH was given ethical approval by NIMR with approval number NIMR/HQ/R.8c/Vol.II/961. The submitted manuscript is part of PhD work within the main project.**

3. Please provide additional details regarding participant consent. In the ethics statement in the Methods and online submission information, please ensure that you have specified (1) whether consent was informed and (2) what type you obtained (for instance, written or verbal, and if verbal, how it was documented and witnessed). If your study included minors, state whether you obtained consent from parents or guardians. If the need for consent was waived by the ethics committee, please include this information.

**Thank you very much for the comment. The study used de-identified routine data from the health facility where participants were being managed for HIV. The ethical committee did not need consent form to be signed by the study participants. Hence, the need for consent was waived by the KCMUCo IRB.**
