# Supplementary material for: Impact of Isoniazid Preventive Therapy on Tuberculosis incidence among people living with HIV: A secondary data analysis using Inverse Probability Weighting of individuals attending HIV care and treatment clinics in Tanzania
Source: PLoS One. 2021 Jul 13;16(7):e0254082. doi: 10.1371/journal.pone.0254082 (PMC8277069; doi:10.1371/journal.pone.0254082)
Supplement: Attachment — Submitted filename: Peer review comments PONE-D-20-37236.docx [file pone.0254082.s003.docx]

Thank you very much for allowing me to review this interesting manuscript.

The manuscript adds to the existing evidence on the effectiveness of IPT in reducing TB incidence based on analysis of a large number of participants in Tanzania. The papers showed durable protection against TB for 6 years. The manuscript is generally well written but lacks some details. Please consider suggestions below to improve clarity.

1. The definition of the IPT group and the control group is not clear to me. The authors conducted the survival analysis by defining entry time as the data of enrolment. Were participants classified into either IPT group or the control group depending on whether they started IPT at the time of enrolment? I presume that most of PLHIV started at IPT the time enrolment but some might have initiated it later (e.g. after investigation for TB). How did you account for those who started IPT later? For example, if they started IPT one month later, were they then transferred into the IPT group? If they remained in the control group, then the control group is not “those who never received IPT”.

2. What is the definition of TB disease? Only bacteriologically confirmed? Does this include TB diagnosed regardless of the timing after the follow-up? (e.g. TB diagnosed within one week after the enrolment.)

3. Figure 2 shows a sharp increase in TB very early in the follow-up. If I understand correctly, participants who screened positive didn’t start immediately upon enrolment and were included in the control group. Among those who screened positive, prevalent TB may have been found after investigation and inflated the number of TB cases in the control group in the initial period.

4. The authors claim that baseline characteristics were balanced after IPTW. However, I seem to find some imbalances, for example:

ART 29.02% vs 36.09%

Private facility 17% vs 29%

Underweight 15% vs 18%

Is this the reason why the authors conducted multivariable cox-regression after IPTW?

I wonder if the authors conducted balance diagnostics. Presenting results may be helpful. <https://www.ncbi.nlm.nih.gov/pmc/articles/PMC4626409/>

I would also suggest presenting the distribution of the weights.

**Minor points**

**Introduction**

Suggest mentioning the coverage of IPT among PLHIV not only the proportion of CTC providing TPT.

Also, suggest to briefly summarize the durability of efficacy reported in high TB burden countries and discuss how the present research adds to the evidence.

Method:

Is TST recommended at all?

If I remember correctly, the TB guidelines in Tanzania recommend repeating IPT after 2 years. Is there any chance that some of the participants repeated IPT?

**Data collection**

How did you assess nutritional status? What is the definition of each category?

How is ambulatory different from walking?

Do you have information on the previous history of TB?

**Analysis**

Please justly the use of p-values less than 0.05 as a criterion to select covariates included in the final model. This may miss important variables associated with exposure and outcome. Have you considered a higher threshold? <https://pubmed.ncbi.nlm.nih.gov/8256780/>

How did you handle missing data?

How were the weights given? Was it simple inverse or did you use stabilized weights?

**Result**

Paragraph 2

“from private health facilities (84.36%).” This is not consistent with Table 1. 84.36% is from public facilities.

Paragraph 3

Suggest reporting the median duration of follow-up in the two groups. The person-years reported don’t seem to correct. Only 134.56 person-year? Is this for all participants or 2309 TB patients? Either way, the number is too small, corresponding to a very short period of follow-up. Person-years in Table 2 are also too small.

Did you find any difference in the number of people who were lost to follow-up or died between the two groups? How could that impact the analysis?

**Discussion**

Paragraphs 2 and 3 seem repetitive.

The discussion could also mention the durability of protection.

Table 1.

Proportions of the weighted sample enrolled by year in the IPT group do not sum up to 100%.
