# Supplementary material for: Impact of Isoniazid Preventive Therapy on Tuberculosis incidence among people living with HIV: A secondary data analysis using Inverse Probability Weighting of individuals attending HIV care and treatment clinics in Tanzania
Source: PLoS One. 2021 Jul 13;16(7):e0254082. doi: 10.1371/journal.pone.0254082 (PMC8277069; doi:10.1371/journal.pone.0254082)
Supplement: Attachment — Submitted filename: plos one comments.docx [file pone.0254082.s004.docx]

**Comments on the plos one paper**

The paper attempted to study a very important question, well done for the initiative. Please find my following comments regarding the article below.

**Abstract**

**Background**: there is no statement that explains the magnitude of the problem (statement of the problem).

**Methods**: study setting and design not included on the methods section of the abstract, when are you going to say the IPT has no effect, reduces the effect or increases the effect, the decision criteria is not set on this section. Or the cut off point is not placed here. Try to include it. In the methods of the abstract, some of the sections of the method of the abstract does not make sense it needs revision.

In the result of the abstract, one change that should be made revolves around the Results sections on the "incidence" is that incidence or prevalence??

**Data collection**: the data collection technique is not included on the data collection section try to include it there.

**Methods section:**

Please clearly elaborate the following procedures: inclusion and exclusion criteria, sampling, methods for data capture and quality control. Any attempt to reduce internal and external biases, which confounders were considered? There is missing of eligibility criteria, Data collection, Handling and Tracking of Missing data, and Quality control

**Ethical consideration**

There is no statement on the ethical consideration that explains how to keep the confidentiality of the participants.try to include a statement.

**Discussion**

You should include a paragraph in the discussion about the significance of your results and how this will inform preventative strategies in the future.
